# Thermal ecological physiology of native and invasive frog species: do invaders perform better?

**DOI:** 10.1093/conphys/cow056

**Published:** 2016-11-18

**Authors:** Pablo A. Cortes, Hans Puschel, Paz Acuña, José L. Bartheld, Francisco Bozinovic

**Affiliations:** 1 Departamento de Ecología, Center of Applied Ecology & Sustainability (CAPES), Facultad de Ciencias Biológicas, Pontificia Universidad Católica de Chile, Santiago 6513677, Chile.; 2 Departamento de Ciencias Básicas, Universidad Santo Tomás, Santiago, Chile.; 3 Instituto de Ciencias Ambientales y Evolutivas, Facultad de Ciencias, Universidad Austral de Chile, Campus Isla Teja, Valdivia, Chile.

**Keywords:** Acclimation, amphibians, central Chile, invasive species, thermal performance curve, thermal tolerance

## Abstract

Biological invasions are recognized as an important biotic component of global change that threatens the composition, structure and functioning of ecosystems, resulting in loss of biodiversity and displacement of native species. Although ecological characteristics facilitating the establishment and spread of non-native species are widely recognized, little is known about organismal attributes underlying invasion success. In this study, we tested the effect of thermal acclimation on thermal tolerance and locomotor performance in the invasive *Xenopus laevis* and the Chilean native *Calyptocephalella gayi*. In particular, the maximal righting performance (μ_MAX_), optimal temperature (*T*_O_), lower (CT_min_) and upper critical thermal limits (CT_max_), thermal breadth (*T*_br_) and the area under the performance curve (AUC) were studied after 6 weeks acclimation to 10 and 20°C. We observed higher values of μ_max_ and AUC in *X. laevis* in comparison to *C. gayi*. On the contrary, the invasive species showed lower values of CT_min_ in comparison to the native one. In contrast, CT_max_, *T*_O_ and *T*_br_ showed no inter-specific differences. Moreover, we found that both species have the ability to acclimate their locomotor performance and lower thermal tolerance limit at low temperatures. Our results demonstrate that *X. laevis* is a better performer than *C. gayi*. Although there were differences in CT_min_, the invasive and native frogs did not differ in their thermal tolerance. Interestingly, in both species the lower and upper critical thermal limits are beyond the minimal and maximal temperatures encountered in nature during the coldest and hottest month, respectively. Overall, our findings suggest that both *X. laevis* and *C. gayi* would be resilient to climate warming expectations in Chile.

## Abstract (precis)

Biological invasions are an important threat to biodiversity. We studied the effect of thermal acclimation on thermal tolerance and locomotor performance in the invasive *Xenopus laevis* and the Chilean native *Calyptocephalella gayi*. We show that *X. laevis* is a better performer than *C. gayi*. Furthermore, thermal tolerance did not differ between the species.

## Introduction

Biological invasions are recognized as an important biotic component of global change (Richardson *et al*., 2000; Ricciardi, 2007; Lockwood *et al*., 2013). Invasive species alter the composition, structure and functioning of ecosystems, resulting in loss of biodiversity and displacement of native species (Talley *et al*., 2001; Rochlin *et al*., 2013). An emergent concern is that other components of global change, such as climate warming, might enhance the capacity of alien species to invade new areas (Dukes and Mooney, 1999; Stachowicz *et al*., 2002; Hellmann *et al*., 2008; Rahel and Olden, 2008; Walther *et al*., 2009; Robinet and Roques, 2010). Lejeusne *et al*. (2014) proposed that both components ‘constitute a deadly-duo threatening species abundance, distributions and biotic interactions’.

At present, however, the organismal attributes that make invading species ecologically successful, in comparison to a native, remain as a poorly answered question (Rejmánek and Richardson, 1996; Snyder and Evans, 2006; Devin and Beisel, 2007). The successful establishment and spread of invasive species in a recipient environment would be facilitated by the ability of an invasive species to maintain high physiological performance over a wide range of environmental conditions (i.e. generalist behaviour; Marvier *et al*., 2004; Snyder and Evans, 2006; Angilletta, 2009; Knop and Neusser, 2012). Nevertheless, among native species a higher performance is typically constrained to a narrow range of conditions (i.e. specialist). As temperature has profound effects on organismal functions, this biotic environmental factor is gaining attention as a major driver of invasion success.

In this context, Kelley (2014) proposed, tested and provided evidence supporting multiple hypotheses to untangle the role that thermal physiology plays in species invasion. Indeed, this author hypothesized that the ability to maintain physiological function across an extensive range of temperature tolerances (i.e. eurythermality) might explain the success of invasive species over native ones (‘greater eurythermal hypothesis’; see Lockwood and Somero, 2011; Zerebecky and Sorte, 2011; Tepolt and Somero, 2014). In addition, it has been proposed that acclimation to higher temperatures is associated with broader thermal tolerance in invasive species (Braby and Somero, 2006; Chown *et al*., 2007; Slabber *et al*., 2007; Hoffmann and Todgham, 2010; Tepolt and Somero, 2014).

The Chilean frog *Calyptocephalella gayi* is an endemic aquatic species inhabiting central Chile (Donoso-Barros and Cei, 1962; Vélez, 2014). This species is the only representative of the genus *Calyptocephalella* and is sometimes referred to as a living fossil (Pyron and Wiens, 2011). *Calyptocephalella gayi* is currently restricted to deep ponds and small water reservoirs in central Chile (Veloso and Navarro, 1988; Rabanal and Nuñez, 2008; Muzzopappa and Nicoli, 2010; Veloso *et al*., 2010). This species is classified as vulnerable by the IUCN (Veloso *et al*., 2010), and current evidence indicates that populations of *C. gayi* are declining in its native range (Glade, 1983; Díaz-Páez and Ortiz, 2003). Moreover, the Chilean frog has been declared as a protected species by the Chilean government, who have prohibited its capture (Glade, 1983). The extensive alteration of Chile's temperate and water regime that have occurred over the last decade plays a key role in creating adverse abiotic conditions for amphibian fauna (Diaz *et al*., 2007; Gutiérrez *et al*., 2012). More importantly, this species is also threatened by the introduction of the aquatic African clawed frog, *Xenopus laevis* (Lobos and Measey, 2002; Lobos and Jaksic, 2005). *Xenopus laevis* is probably one of the invasive amphibian species with the greatest worldwide distribution (Lowe *et al*., 2000; Tinsley *et al*., 2015). This generalist predator was introduced into Chile in the early 1980s, occurring in a very wide range of habitats, including those occupied by *C. gayi* (Glade, 1983; Veloso and Navarro, 1988). In fact, since its invasion in Chile, this species has colonized an area of ~21 200 km^2^ (Lobos and Jaksic, 2005). Currently, there is scarce knowledge on the factors and processes underlying its distributional pattern.

It is surprising to see that, despite the substantial negative impact of the African clawed frog on the Chilean frog populations, the basic organismal traits of what makes *X. laevis* successful in comparison to *C. gayi* are practically unknown. This is particularly relevant in times when climate is changing more rapidly than an amphibian can adjust (Pörtner and Farrell, 2008; Bozinovic and Pörtner, 2015).

Locomotor performance is an ecologically relevant parameter because it affects the ability of an organism successfully to reproduce, forage, escape predators or disperse into novel habitats (Garland and Losos, 1994; Higham, 2007; Husak and Fox, 2008; Seebacher and Franklin, 2011). Extreme environmental temperatures can affect muscle contractile properties and enzyme activities and, consequently, locomotor performance and survival (Bennett, 1990; Navas, 1996; Johnston and Temple, 2002; Herrel and Bonneaud, 2012; Careau *et al*., 2014). A rich history of research on the ability of ectothermic vertebrates to make adjustments that alter their locomotor performance after exposure to different thermal regimes has revealed that acclimation at low and moderate temperatures tends to improve locomotor performance. At higher temperatures, in contrast, locomotor performance is decreased (see review and examples in Navas *et al*., 2008 and references therein). These studies also have reported that acclimatory responses of locomotor performance take several weeks to occur (Wilson and Franklin, 1999; Wilson *et al*., 2000). The success of invasive species may also be influenced by the thermal dependence of locomotor performance. In particular, the capacity for invasion and range expansion of most ectothermic vertebrates relies upon active dispersal, which in turn can be influenced by thermal effects on physiological mechanisms that support locomotor performance (Seebacher and Franklin, 2011; McCann *et al*., 2014; Winwood-Smith *et al*., 2015).

Among the locomotor performance traits that can be studied in reptiles and amphibians, the righting response (i.e. the time it takes an individual to return to a prone position after being placed upside down) has received particular attention because it is rather easy to quantify in experimental conditions, shows a strong thermal dependence, can be related directly to muscle physiology and is related to fitness (John-Alder *et al*., 1988; Lutterschmidt and Hutchison, 1997; Steyermark and Spotila, 2001; Freedberg *et al*., 2004; Elnitsky and Claussen, 2006; Delmas *et al*., 2007). Moreover, the temperature at which animals are incapable of a coordinated locomotor response and their righting response is lost has been used as an indicator of tolerance to extreme temperatures in anuran amphibians (John-Alder *et al*., 1988; Lutterschmidt and Hutchison, 1997; Herrel and Bonneaud, 2012).

In this study, we tested the effect of thermal acclimation on the comparative thermal tolerance and locomotor performance in the invasive *X. laevis* and the native *C. gayi*, in central Chile. We use these data to address the following two questions. (i) Does the African clawed frog perform better than the Chilean frog? (ii) Are African clawed frogs more eurythermal than Chilean frogs? If the hypothesis of a greater eurythermality of invasive species is supported (Zerebecki and Sorte, 2011; Barahona-Segovia *et al*., 2016) then we predict that African clawed frogs will exhibit higher and broader performance than the native Chilean frogs, i.e. the invasive species will have a broader physiological tolerance than the geographically overlapping native species (Kelley, 2014).

## Materials and methods

### Experimental animals

Ten adult individuals of *C. gayi* and *X. laevis* were purchased from an animal breeding store in Central Chile. While held captive, animals were maintained in a natural thermal regime. Animals were transported to our laboratory and placed in individual plastic cages of 45 cm (length) × 30 cm (width) × 20 cm (height) filled with water and fed with beef liver. All plastic cages were maintained inside a climatic chamber (Pitec). The Universidad Católica animal care committee approved all experimental procedures.

### Thermal acclimation and measurements of the righting response

Before experimental procedures, individuals of both species were randomly divided into two groups (10 specimens for each species; five subjected to treatment and five to control conditions). The first group was maintained at 20 ± 1°C (warm acclimation) in a climatic chamber (Pitec), while the second group was maintained at 10 ± 1°C (cold acclimation). These temperatures were chosen because they represent average environmental temperatures experienced by *X. laevis* and *C. gayi* during summer and winter, respectively (meteorological database obtained from the Chilean government agency Dirección General de Aguas). All groups were acclimated for 6 weeks and kept with a photoperiod of 12 h light–12 h dark. This period is in accordance with previous studies performed in *X. laevis* and other frog species (Wilson and Franklin, 1999; Wilson *et al*., 2000).

**Figure 1:**
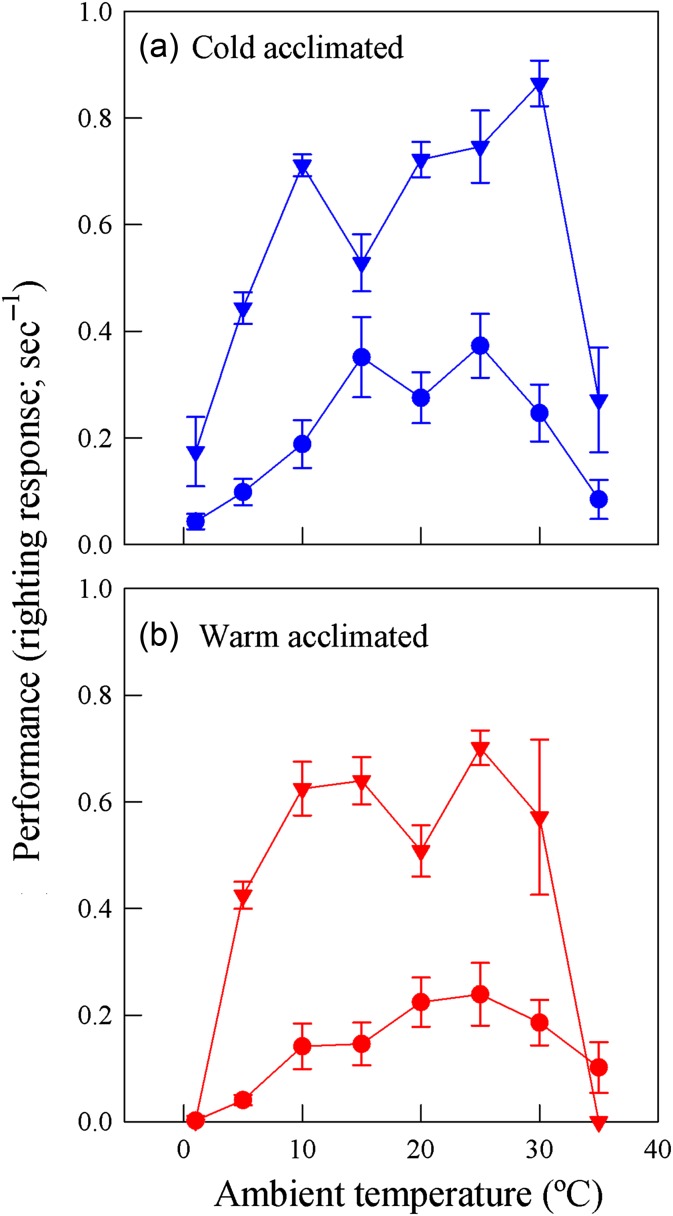
Performance curves of righting response in both the native *Calyptocephalella gayi* (circles) and the invasive *Xenpus laevis* (triangles) acclimated for 6 weeks at 10°C (cold acclimation; **a**) and 20°C (warm acclimation; **b**). There were 10 specimens of each species; five subjected to treatment and five to control conditions. Values are shown as means ± SEM.

After acclimation, we measured the righting response, i.e. the time it takes an individual to return to a prone position after being placed upside down, in four groups at eight different temperatures during eight consecutive days. We used the righting response as a proxy of locomotor performance, because it is rather easy to quantify in experimental conditions, shows a strong thermal dependence, is related to fitness and, more importantly, is an extremely safe method that does not compromise the animal's integrity. The experimental protocol was as follows. At 10.00 h, animals were exposed to the experimental temperature. At 18.00 h, animals were placed in a temperature-controlled room at 20 ± 1°C, and righting response measurements were performed immediately (<2 min). The procedure outlined above was repeated for eight consecutive days, and the sequence for experimental temperatures was as follows: day 1, 20 ± 1°C; day 2, 5 ± 1°C; day 3, 15 ± 1°C; day 4, 25 ± 1°C; day 5, 10 ± 1°C; day 6, 30 ± 1°C; day 7, 35 ± 1°C; and day 8, 1 ± 1°C (Fig. 1). Body mass (*m*_b_) was measured prior to all experimental proceedings with an electronic balance (Sartorius PT-600 Portable Digital Precision Balance; precision, ±0.01 mg).

### Data analysis

To address our questions, data on maximal righting performance (μ_MAX_), optimal temperature (*T*_O_), which represents the temperature at μ_MAX_, lower (CT_min_) and upper critical thermal limits (CT_max_), thermal breadth (*T*_br_) and the area under the performance curve (AUC) were computed by adjusting the righting response data to a second-order polynomial function using the free software CurveExert professional 2.0. The performance curve breadth can be determined when performance goes to zero at either extreme. Also, the AUC is an indicator of the total integrated individual's performance and can be estimated by integrating the polynomial function.

The effect of thermal acclimation and species on all variables curve was tested by two-way analysis of covariance using structural body size as a covariate, with temperature and species as fixed factors. Structural body size was estimated by a combination of multiple morphometric measurements (body mass, head–cloaca length and body width) using a principal component analysis, and we used the first component as our measure of structural body size. The μ_MAX_ data were normalized with respect to the maximal value in the database. Normality and homoscedasticity of data were tested using the Kolgomorov–Smirnov and Cochrane C test, respectively. When differences were significant (*P* < 0.05), we used the *a posteriori* Tukey test for multiple comparisons. Data analysis was performed using Statistica 6.1 (2006), and results are shown as means ± SEM.

## Results

Summary statistics for the thermal physiological traits obtained from the thermal performance curves are presented in Table 1.

**Table 1:** Descriptive statistics (means ± SEM) of the studied variables in the invasive African clawed frog (*Xenopus laevis*) and the native the Chilean frog (*Calyptocephalella gayi*)

	*Xenopus laevis*		*Calyptocephalella gayi*	
Variable	10°C	20°C	10°C	20°C
*m*_b_ (g)	59.36 ± 5.98	68.14 ± 5.39	66.54 ± 3.13	58.94 ± 2.33
μ_max_ (s^−1^)	0.80 ± 0.03	0.75 ± 0.01	0.48 ± 0.12	0.21 ± 0.05
*T*_o_ (°C)	20.02 ± 0.78	18.27 ± 0.40	20.79 ± 1.11	22.26 ± 1.58
CT_min_ (°C)	−1.95 ± 1.01	0.42 ± 0.21	1.06 ± 0.32	1.77 ± 0.44
CT_max_ (°C)	41.84 ± 2.24	36.21 ± 0.69	40.42 ± 2.38	42.72 ± 3.01
*T*_br_ (°C)	43.79 ± 3.09	35.78 ± 0.64	39.36 ± 2.55	40.95 ± 2.93
AUC	23.49 ± 2.13	17.97 ± 0.52	11.97 ± 2.50	5.86 ± 1.35

There were 10 specimens of each species; five subjected to treatment and five to control conditions. Abbreviations: AUC, total area under the thermal tolerance curve; CT_min_, lower critical thermal limit; CT_max_, upper critical thermal limit; *m*_b_, body mass; μ_max_, maximal righting performance; *T*_br_, breadth of the performance curve; and *T*_O_, temperature at which performance is maximized.

### Righting response, optimal temperature and area under the thermal tolerance curve

Maximal performance or μ_MAX_ was affected by species (*F*_(1,15)_ = 25.23, *P* < 0.01) and acclimation temperatures (*F*_(1,15)_ = 5.02, *P* < 0.05), but not by the interaction between species and acclimation temperatures (*F*_(1,15)_ = 2.00, *P* = 0.18). *Xenopus laevis* had higher μ_MAX_ than *C. gayi* regardless of the acclimation temperature. The effect of temperature, however, differed between species. Indeed, cold-acclimated *C. gayi* had higher μ_MAX_ compared with warm-acclimated animals, whereas *X. laevis* maintained μ_MAX_ independent of thermal treatment. In contrast, *T*_O_ did not differ between species (*F*_(1,15)_ = 1.96, *P* = 0.18) or temperature (*F*_(1,15)_ = 0.00, *P* = 0.98), nor by the interaction between species and temperature (*F*_(1,15)_ = 1.07, *P* = 0.32).

Regarding AUC, there was a significant effect of species (*F*_(1,15)_ = 31.90, *P* < 0.01) and temperature (*F*_(1,15)_ = 8.79, *P* < 0.01), but not an effect of the interaction between species and temperature (*F*_(1,15)_ = 0.18, *P* = 0.68). Independently of the acclimation temperature, the invasive *X. laevis* had the greater AUC in comparison to the native *C. gayi*. The area enclosed by the thermal tolerance curve in both species was greater in cold-acclimated than in warm-acclimated frogs.

### Thermal tolerance limits and the performance curve breadth

The lower critical thermal limit was significantly affected by species (*F*_(1,15)_ = 15.31, *P* < 0.01) and acclimation temperatures (*F*_(1,15)_ = 5.61, *P* < 0.05). The interaction of species and temperature had no significant effect on CT_min_ (*F*_(1,15)_ = 0.53, *P* = 0.48). The alien species always exhibited lower CT_min_ values than *C. gayi* in both cold and warm thermal treatments. Also, CT_min_ in frogs acclimated to 10°C was significantly lower than in those acclimated to 20°C. On the contrary, there were not significant effects of species (*F*_(1,15)_ = 0.11, *P* = 0.74) or acclimation temperature (*F*_(1,15)_ = 0.26, *P* = 0.62) nor of the interaction between species and acclimation temperature (*F*_(1,15)_ = 1.29, *P* = 0.27) on CT_max_. Like CT_max_, *T*_br_ was not affected by species (*F*_(1,15)_ = 0.36, *P* = 0.56), temperature (*F*_(1,15)_ = 1.01, *P* = 0.33) or by the interaction between species and temperature (*F*_(1,15)_ = 1.46, *P* = 0.25).

## Discussion

This study addressed an important question: does the alien invasive species, *X. laevis*, perform better than the co-habiting native species, *C. gayi*? (Daehler, 2003; Jumbam *et al*., 2008; Van Kleunen *et al*., 2010; Anacleto *et al*., 2014; Barahona-Segovia *et al*., 2016). Moreover, there is increasing evidence that climate warming might also influence the dynamics of biological invasion (Dukes and Mooney, 1999; Stachowicz *et al*., 2002; Hellmann *et al*., 2008; Walther *et al*., 2009; Robinet and Roques, 2010). We evaluated the effect of thermal acclimation on thermal tolerance and locomotor performance in the invasive *X. laevis* and the native living fossil *C. gayi*, two aquatic amphibian species inhabiting the same habitats in central Chile.

Within this context, our key findings were higher values of μ_max_ and AUC in the invasive species in comparison to the native one. In contrast, *X. laevis* showed lower values of CT_min_ in comparison to *C. gayi*. The values of CT_max_, *T*_O_ and *T*_br_ showed no inter-specific differences. Moreover, we found that both *X. laevis* and *C. gayi* have the ability to acclimate their locomotor performance and lower thermal tolerance limit at low temperatures.

### Is the African clawed frog a better performer than the Chilean frog?

It is widely recognized that invasive species not only have higher values for traits associated with performance than non-invasive species, but also have the ability to maintain high performance over a wide range of environmental conditions (Daehler, 2003; Burns, 2004; Van Kleunen *et al*., 2010; Barahona-Segovia *et al*., 2016; Seebacher *et al*., 2015; Winwood-Smith *et al*., 2015). Current evidence suggested that traits linked to locomotor performance might facilitate a rapid expansion of the invading populations in both terrestrial and aquatic species (Phillips *et al*., 2006; Llewelyn *et al*., 2010; Niewiarowski *et al*., 2012; Polo-Cavia *et al*., 2012). Previous studies on amphibians have reported that the accelerating rate of cane toad (*Rhinella marina*) invasion through tropical Australia is correlated with enhanced locomotor abilities (Phillips *et al*., 2006; Llewelyn *et al*., 2010). In agreement with this, our results showed higher values of maximal locomotor performance in the invasive African clawed frog in comparison the native Chilean frog. Interestingly, we did not observe inter-specific differences in *T*_O_. Indeed, amphibians acclimated to 10°C had *T*_O_ values (*X. laevis*, 20.0 ± 0.78°C; and *C. gayi*, 20.79 ± 1.11°C) that were generally higher than the mean temperature encountered by these species in nature during the winter (June–August 2015, 12.41°C). This finding would indicate that both species have the capacity to tolerate colder thermal conditions during the winter by physiological plasticity. We also observed that *T*_O_ values of *X. laevis* acclimated to 20°C (18.27 ± 0.27°C) were markedly closer to the mean temperature encountered in nature during summer in central Chile (April 2015, 18.39°C). In contrast, cold-acclimated *C. gayi* (22.26 ± 1.11°C) exhibited generally higher *T*_O_ values than summer temperatures. This result suggests that *X. laevis* currently lives in a thermal environment were warm tolerance is maximized and, putatively, will be vulnerable to the predicted temperature increment in Central Chile (2–4°C; Fuenzalida *et al*., 2007; Cabré *et al*., 2010).

Recent theoretical work by Palaima (2007) suggests that the AUC may act as an indicator of the total integrated individual's performance. In addition to the maximal value of performance and the breadth of the curve, comparisons of AUC among species also provide very valuable information to distinguish a generalist from a specialist species (Gilchrist, 1995; Gvoždík and Van Damme, 2008). Theoretically, a thermal generalist can persist in a wide range of thermal conditions and, therefore, may have a broader tolerance range than a specialist. However, a generalist will have also a much lower maximal performance than a specialist (Gilchrist, 1995, 1996; Angilletta *et al*., 2003). Palaima (2007) also states that a thermal generalist would have higher values of AUC compared with the specialist.

Recent evidence suggests that invasive species are more likely to be generalists than specialists, and thus more successful than natives in adapting or acclimating to new habitats (Moyle and Marchetti, 2006; Evangelista *et al*., 2008). Our results showed that: (i) *X. laevis* exhibited a higher AUC in comparison to the native *C. gayi*; (ii) *X. laevis* exhibited higher values of maximal locomotor performance in comparison the native *C. gayi*; and (iii) the alien and native frogs did not display differences in tolerance breadth. Overall, our findings did not provide strong evidence that the alien *X. laevis* exhibits a ‘thermally generalist’ strategy, but demonstrated that *X. laevis* always performed better than *C. gayi* over the entire range of temperatures evaluated in this study.

Time use and activity of frogs and other ectotherms depends on abiotic factors, such as temperature. An acute decrease in temperature results in lower body temperatures, reduced contraction velocity of muscles and, ultimately, lower locomotor performance (Christian and Tracy, 1981; Else and Bennett, 1987; Navas *et al*., 1999; Herrel and Bonneaud, 2012; Sun *et al*., 2014). In the present study, μ_MAX_ and AUC of both *X. laevis* and *C. gayi* acclimated to 10°C were higher than those of animals acclimated at 20°C. Our findings are in agreement with previous studies showing that African clawed frogs inhabiting Europe have the ability to compensate for the effects of lower temperatures by enhancing their swimming performance (Wilson *et al*., 2000). Moreover, our results are in accordance with the ‘colder is better’ hypothesis, which predicts that organisms acclimated to low temperatures could achieve greater locomotor performance than others held in warm conditions (Deere and Chown, 2006; Frazier *et al*., 2008; Esterhuizen *et al*., 2014).

### Are the African clawed frogs more eurythermal than the Chilean frogs?

Previous evidence suggested that both CT_min_ and CT_max_ are highly flexible traits that respond to changes in temperature (Li *et al*., 2009; Chanthy *et al*., 2012; Ruiz-Aravena *et al*., 2014; Davies *et al*., 2015). As we mentioned above, there are higher inter-specific differences and variability in CT_min_, in comparison to CT_max_ and *T*_br_, among *X. laevis* and *C. gayi*. The CT_min_ of cold-acclimated *X. laevis* was generally below the minimal temperature encountered by these species in nature during the coldest month (June 2015, −2.0°C) in Central Chile. In contrast, the CT_min_ of both cold- and warm-acclimated *C. gayi* were generally above the minimal temperature encountered in nature. The CT_max_ of both species was generally above the maximal temperature encountered in nature during the hottest month (April 2015, 37.8°C).

Lobos *et al*. (2013) made a bioclimatic niche model considering areas susceptible to invasion. From the habitat projection in northern and southern South America, they predict high habitat suitability for *X. laevis* in Chile. Our results on cold tolerance are in accordance with the projected expansion of *X. laevis* in Chile, where it is expected that *X. laevis* will have a higher probability to survive, invade and establish in colder places in Chile (Lobos *et al*., 2013). In this context, the physiological analyses used in our study could provide new and valuable information to be considered in species invasions using ecological niche modelling, and thus improve our understanding of areas susceptible to invasion by *X. laevis*. This is particularly important under a global warming scenario, because the temperature is expected to increase as much as 2–4°C in central and southern Chile by 2050 (Fuenzalida *et al*., 2007; Cabré *et al*., 2010). Hence, our findings suggest that both the invasive *X. laevis* and the native *C. gayi* could be resilient to climate warming expectations in Chile.

In the present study, we found that *X. laevis* and the native *C. gayi* were able to change the lower, but not the higher, thermal limit after 6 weeks of acclimation. This finding provides support for the idea that physiological adjustments to cold and heat stress are at least uncoupled in ectotherms (Klok and Chown, 2003; Jumbam *et al*., 2008; Lalouette *et al*., 2012; Hoffmann *et al*., 2013; Bozinovic *et al*., 2014; Davies *et al*., 2015). For example, Araújo *et al*. (2013) show a direct and positive relationship between lower thermal limits and environmental temperature in amphibians. Nevertheless, recently Bozinovic *et al*. (2016) reported that flies acclimated to environments with changing thermal variance reduce the scope of thermal tolerance. Furthermore, they observed that the heat tolerance of flies seems to be more conserved with lower variation among acclimation treatments. Nevertheless, this is not exact in flies acclimated to a scenario of changing mean with changing variance, where flies exhibited significantly lower CT_max_ values. This could be explained by the fact that the physiological mechanisms underlying cold and warm tolerance could be different. Ectotherms would tolerate high temperatures by increasing the expression of genes related to protein folding as well as chaperones and proteasome proteins (Gehring and Wehner, 1995; Healy *et al*., 2010; Zhang *et al*., 2015), whereas the expression of cryoprotectants and antifreeze proteins is associated with cold tolerance (Constanzo and Lee, 2013; Long *et al*., 2013; Kawarasaki *et al*., 2014; Zhang *et al*., 2015).

Invasive species are often assumed to establish and spread in a new habitat as a consequence of their ability to make physiological adjustments that maintain performance across an extensive range of environmental conditions. As pointed out before, the greater eurythermal hypothesis predicts that invasive species have a broader physiological tolerance than native species occupying the same habitat (Zerebecki and Sorte, 2011; Kelley, 2014). Today, many studies support this hypothesis in both terrestrial and aquatic ectotherms (Zerebecki and Sorte, 2011; Yu *et al*., 2012; Bates *et al*., 2013; Lejeusne *et al*., 2014; Tepolt and Somero, 2014). In fact, a meta-analysis performed by Kelley (2014) revealed that a higher temperature tolerance was positively related to the thermal width for invasive but not native species. Our results show that although there are differences in the lower thermal critical limit, the invasive and native frogs did not differ in their thermal tolerance, rejecting the greater eurythermal hypothesis in this case. These results, however, must be viewed with caution, because locomotion is a whole-organism functional trait whose expression requires elaborate biochemical and metabolic integration. Hence, the establishment and spread of invasive species into new habitats can be explained by adjustments of the physiological systems supporting locomotion (Lefrançois *et al*., 2005; Cano and Nicieza, 2006; Anacleto *et al*., 2014). For example, Seebacher and Franklin (2011) documented that mitochondrial ATP production capacity could constrain the advance of cane toads to cooler southern areas of Australia. Finally, we emphasize the importance of future studies that consider other physiological traits to understand the mechanisms that underlies the thermal differences in performance between the invasive *X. laevis* and the native *C. gayi*, two frog species inhabiting central Chile.

## Funding

This work was supported by the Fondo Nacional de Desarrollo Científico y Tecnológico (FONDECYT-3150215 to P.A.C.; FONDECYT-3140243 to J.L.B.) and Center of Applied Ecology & Sustainability (CAPES FB002-2014 line 3 to F.B.).
